# The “*Journal of Functional Morphology and Kinesiology*” Journal Club Series: Highlights on Recent Papers in Corrective Exercise

**DOI:** 10.3390/jfmk5040074

**Published:** 2020-10-14

**Authors:** Antonino Bianco, Silvio Lorenzetti, Jan Seiler, Heiliane de Brito Fontana, Walter Herzog, Gessica Aline Silvano, Heron Baptista de Oliveira Medeiros, Giuseppe Musumeci

**Affiliations:** 1 Department of Psychology, Educational Science and Human Movement, University of Palermo, Via Giovanni Pascoli, 6, 90144 Palermo, Italy; antonino.bianco@community.unipa.it; 2 Swiss Federal Institute of Sport Magglingen (SFISM), 2532 Magglingen, Switzerland; sl@ethz.ch (S.L.); jan.seiler@baspo.admin.ch (J.S.); 3 Biomechanics Laboratory, Federal University of Santa Catarina, Florianopolis 88040400, Brazil; heiliane.fontana@ufsc.br (H.d.B.F.); Gessicasilvano2013@gmail.com (G.A.S.); heronbomed@gmail.com (H.B.d.O.M.); 4 Faculty of Kinesiology, Engineering, Medicine and Veterinary Medicine, University of Calgary, Calgary, AB T2N 1N4, Canada; wherzog@ucalgary.ca; 5 Department of Biomedical and Biotechnological Sciences, Anatomy, Histology and Movement Sciences Section, School of Medicine, University of Catania, Via S. Sofia 87, 95123 Catania, Italy; 6 Research Center on Motor Activities (CRAM), University of Catania, 95123 Catania, Italy; 7 Department of Biology, College of Science and Technology, Temple University, Philadelphia, PA 19122, USA

**Keywords:** corrective exercise, therapeutic tool, physical fitness, pathology, human movement, rehabilitation, postural disorders, adapted physical activity

## Abstract

We are glad to introduce the Journal Club of Volume Five, fourth Issue. This edition is focused on relevant studies published in the last few years in the field of corrective exercise, chosen by our Editorial Board members and their colleagues. We hope to stimulate your curiosity in this field and to share a passion for sport with you, seen also from the scientific point of view. The Editorial Board members wish you an inspiring lecture.

## 1. Introduction

The theory of using exercise as a therapeutic tool is by no means a new one. As far back as the early nineteenth century, structured exercise was being used for the treatment of sedentary lifestyles, in the form of group gymnastics. By the turn of the twentieth century, the forward-thinking work of Eustace Miles and Eugene Sandow inspired many prominent physiologists and physicians to become interested in the use of exercise as a therapeutic modality [1]. The large use of anthropometric measurements at the time introduced evaluation protocol into the exercise arena. As the mid-twentieth century approached, the growing acceptance of the psychological benefits of exercise led to the introduction of mind–body exercise systems. The efforts of prominent physical educators, such as Frederick Mathias Alexander, Moshe Feldenkrais, Joseph Pilates and Milton Trager were instrumental in this development. Mainly provoked by their own personal experiences of major illness or musculoskeletal impairment, they documented the intimate relationship between physical fitness and pathology. Essential to their approach was an explicit understanding of human movement and how it relates to the efficient functioning of the body. These hypotheses were further supported by the work of Rudolf Laban and Irmgard Bartenieff. Exercise was becoming well-known as a therapeutic tool within the context of physical rehabilitation and the basics for the field of corrective exercise were being laid. Today’s current and habitually inactive lifestyle has reduced the need for spontaneous and functional movement, resulting in a multitude of musculoskeletal dysfunctions. The augmented reputation and use of gyms have motivated much back into the exercise arena. Combined with the growing responsibility that individuals are taking for their own health, this has stimulated increased interest in exercise as a therapeutic tool [2]. As a result, there has been a merging of knowledge from the disciplines of rehabilitation and exercise. The field of corrective exercise bridges the gap between pure rehabilitation and exercise science. Musculoskeletal dysfunction is commonly caused by biomechanical weakness. With this in mind, the use of exercise to correct a dysfunction is an interesting approach to rehabilitation [2]. When exercise training is structured and integrated correctly, using a multifaceted approach, it can help the client/patient to achieve, maintain and enhance their rehabilitative goals, often without the need for other interventions. A well-devised a corrective exercise programme can enhance muscle performance, decrease the severity of the injury, decrease the risk of re-injury and accelerate recovery and return to activity. Long-term solutions should focus not only on correcting the root cause of the problem but also on prevention and teaching optimal movement patterns, for lifelong health and function. Evaluation and exercise program design are at the heart of the successful corrective exercise.

## 2. An Evidence-Based Approach to Hamstring Training

### Highlighted by Jan Seiler

The importance of hamstring strength for horizontal body propulsion, muscle injury prevention and knee stability has recently received a lot of attention [2,3,4]. Athletes capable of producing high horizontal ground reaction force also display high eccentric torque production by the hip extensors (especially hamstrings) at the end of the swing phase of the stride cylce [2]. At the same time, it has been reasoned that athletes are most susceptible to hamstring muscle injury (HMI) at the end of the swing phase, where the hamstrings are operating at a long muscle length (a result of simultaneous hip flexion and knee extension) [5,6]. Furthermore, the hamstring muscles are important contributors to dynamic knee-joint stability in multiplanar movements, and a high level of eccentric hamstring strength seems to have a preventive effect against anterior cruciate ligament (ACL) tears [5].

To train an effective deceleration of the knee extension in sprinting, the focus should be on high-load eccentric contractions at the knee-joint with a flexed hip (≈80°) in order to create an excessive elongation stress. Exercises like the nordic hamstring exercise (NHE), eccentric box drops, single leg deadlifts, eccentric leg curls and eccentric hip extensions meet these criteria [7,8].

Due to the apparent importance of eccentric hamstring strength, the NHE, thanks in part to its simplicity, has become a popular prevention exercise in many sports. The supramaximal characteristics of the NHE have been shown to increase strength and change muscle architecture rapidly (≤4 weeks). Chronic exposure to eccentric training also augments muscle-tendon stiffness, which helps protect a stretching muscle from overload damage [5,6,9,10]. In a recent meta-analysis, van Dyk et al. showed a 51% reduction in HMI in groups training regularly with the NHE [11]. In addition to helping prevent HMI, the exercise shifted the hamstrings’ force–length relationship, which increased the elastic energy recovery during the stretch–shortening cycle at the end of the swing phase, thereby enhancing horizontal propulsive force production in the subsequent foot contact [3,6]. 

Unfortunately, many athletes do not have enough strength to perform the NHE through its full range of motion and reach the break point well before the knee angles at which HMI typically occurs. In order to train greater knee extension angles, either assistance with resistance bands or cable machines or an increase in the slope of the lower leg support is recommended. These methods facilitate training at more specific muscle lengths while also increasing mechanical muscle loading (especially by increasing the time under tension) [12,13,14]. In general, prevention-oriented hamstring exercises performed at longer muscle lengths have been shown to be significantly better than conventional exercises [15]. To approach even more specific hamstring muscle operating lengths, Hegyi et al. suggested a variation of the NHE with 90° of hip flexion. The authors showed that the more pronounced eccentric muscle work achieved in this manner facilitated hamstring fascial elongation, which seems to be crucial for the protective effect [16]. O’Halloran et al. argued that using the Romanian Deadlift Exercise prior to the NHE ellicits slightly more hamstring activation, and is thus recommended as a pre-activation exercise for the NHE. To increase the intensity of the NHE, adding weight or using external impulses (e.g., another person pushing down on the back) have shown to stimulate even greater architectural adaptations [7,17,18]. 

In addition to the general strength level, limb imbalances are also considered a critical factor for injury. As the NHE exceeds the initial strength level of many athletes, a unilateral set-up seems to be rather inappropriate. The sliding leg curl (SLC) offers an interesting alternative to focus on eccentric strength development at long muscle lengths, as well as in a more specific, unilateral manner [19]. Additionally, single-leg supine exercises show a high activation of the gluteus maximus and medius (lumbo-pelvic stabilizers), which seem to have a preventive effect on knee injury (valgus control) and back pain [20]. Furthermore, strong gluteal muscles protect the hamstring muscles from HMI in fatigued conditions [21]. To progressively overload the eccentric part of the SLC, a resistance band can be stretched around the ankles. An eccentric stimulus at higher movement velocities is also possible when the knee extension deceleration is consciously delayed [19]. However, Guex and Millet noted that adaptations in sprint specific eccentric hamstring strength were independent of the exercise movement velocity [7]. 

Verall et al. showed that intervention programs based on exercises that more accurately reflected match playing conditions resulted in a significant reduction in the number of HMIs, and consequently in the number of missed competitions [22]. Mendiguchia et al. stated that high-speed sprinting itself is the most potent stimulus for increasing hamstring fascial length. At the same time, the direct effect on sprint performance and mechanical outcomes is more prominent compared to other interventions, making this approach a “win–win” strategy. However, high-speed sprint training as an injury prevention measure needs careful implementation to ensure both protective and performance effects without elevating the injury risk [23,24,25].

In addition to the HMI paradigm, high eccentric strength of the hamstring muscles seems to play an important role in the prevention of ACL ruptures caused by rapid anterior shift of the tibia in deep knee flexion or a high knee loading movement pattern with dynamic knee valgus loading [26,27]. As ACL injury mechanisms occur within 40 ms, strategies to optimize co-contraction or pre-activation of the thigh muscles seem to be of major importance, even if a preventive effect of such strategies has yet to be shown empirically [28,29]. However, specific neuromuscular training has been shown to induce a change in the pattern of neuromuscular activation of the hamstring muscles during sidecutting maneuvers, and may thereby decrease the risk of dynamic knee valgus movements [30].

A specific multifactorial and individualized hamstring training approach is required for an optimized prevention framework, which should, in the end, improve short-, mid- and long-term performance outcomes [31].

## 3. Corrective Exercises in Elite Sport 

### Highlighted by Silvio Lorenzetti

In elite sport, corrective exercises are mainly used to eliminate athletes’ deficits and imbalances. An elite track and field study (*n* = 121) showed that functional movement screening (FMS), especially with regards to asymmetry, may be crucial for athletes’ development [32]. Functional movement ability, which is associated with the likelihood of future injury, is also related to longitudinal competitive performance outcomes. Campa et al, 2019 [33] recently used FMS to assess dysfunctional, asymmetrical and painful movements in soccer players from four teams. Two teams performed a corrective exercise program and two teams were the control group. There was a high prevalence of asymmetric movements during FMS testing, but movement patterns improved during the competitive season following a specific corrective exercise program. In elite female volleyball players, a 12-week intervention of corrective exercises improved total FMS, shoulder mobility, deep squat, hurdle step, in-line lunge, push up and rotator stability balance scores [34]. Therefore, including corrective exercises in training schedules could reduce injury and enhance performance. Finally, corrective exercises throughout the lifespan should sport-specific to maintain the normal development of the spinal column in athletes [35]. 

Although corrective exercises improve athletes’ asymmetry, imbalances and weaknesses, there is limited literature available on this topic, because cohorts and differences between cohorts are often small, and not all athletes have the same deficits. Furthermore, measuring functional movement is not trivial, and researchers in this area must improve the sensitivity of screening and measurement tools. 

## 4. Obesity, Exercise and Musculoskeletal Disease

### Highlighted by Heiliane de Brito Fontana and Walter Herzog

Obesity is a world-wide problem with no immediate solution, e.g., [36]. Obesity is associated with a number of co-morbidities including diabetes, cardiovascular diseases, metabolic syndrome, and musculoskeletal degeneration, most notably osteoarthritis of weight bearing (hip, knee) and non-weight bearing (finger) joints, e.g., [37]. Aside from joint arthroplasty, exercise has been shown to be among the best strategies not to necessarily stop or reverse osteoarthritis, but to reduce pain and limit medication use, e.g., [38]. Nevertheless, epidemiologic research suggests that few clinicians prescribe exercise to obese patients with mild and moderate osteoarthritis of lower limb joints and will often recommend against exercise in many cases of moderate and severe osteoarthritis, e.g., [39].

Evidence from our lab using a high fat/high sucrose diet induced rat obesity model suggests the following:

A high fat/high sucrose diet results in obesity of the animals (quantified using body fat percentage), and, over the long-term, leads to metabolic syndrome, characterized by chronic systemic (blood) inflammation and decreased insulin sensitivity, and to characteristic changes in the gut microbiota [40]. It results in local (joint) inflammation and causes knee and shoulder joint osteoarthritis [41], plus fibrosis and fat invasion in fast twitch but not slow twitch muscles [42].

Aerobic exercise (30 min of treadmill walking/running five days a week), introduced in parallel with the high fat/high sucrose diet, still results in obesity but reverses many aspects of metabolic disease and normalizes the gut microbiota to control levels. The knee joint osteoarthritis is also prevented [43].

Aerobic exercise (as above) introduced 12 weeks after the onset of the high fat/high sucrose exposure (when knee and shoulder osteoarthritis have reached moderate to severe levels) does not change the progression of osteoarthritis, but normalizes the metabolic syndrome and the gut microbiota [44]. 

Our results support much of what has been reported in the literature. What conclusions might we be able to draw from these series of studies, always noting that these are pre-clinical studies whose translation to the human disease might not be directly possible?

A high fat/high sucrose (a western type diet) is a risk factor for hip and knee osteoarthritis, and in our pre-clinical model, produces hip and knee osteoarthritis quickly and reliably. 

A high fat/high sucrose diet seems to be a risk factor for muscle fibrosis and fat invasion for fast but not for slow twitch fibred muscles, suggesting that aerobic capacity of muscles might be a protector of muscle, and possibly joint disease, caused by obesity and metabolic syndrome.

Exercise in conjunction with a high fat/high sucrose type diet might still lead to obesity (increased body fat percentage compared to normal), but ameliorates metabolic syndrome, insulin sensitivity, and systemic and local inflammation, with the result of healthy knee and hip joints. 

Exercise introduced late following obesity induction and the development of metabolic syndrome does not rescue the knee and hip joints from osteoarthritis or slow down the degeneration but can rescue the metabolic syndrome and associated systemic and local inflammation. 

We suggest that exercise is an excellent strategy for reducing the risk of hip and knee joint osteoarthritis in people with obesity by reducing signs of metabolic syndrome including systemic and local inflammation, insulin sensitivity, and the gut microbiota. We further suggest that in people with obesity and advanced hip and knee osteoarthritis, exercise is beneficial for overall health, improves the quality of life, and reduces the effects of co-morbidities, while not necessarily decreasing the rate of osteoarthritis progression. 

## 5. Hip Biomechanics, Strength, and Lower Limb Injuries

### Highlighted by Heiliane de Brito Fontana, Walter Herzog, Gessica Aline Silvano and Heron Baptista de Oliveira Medeiros

“Excessive” femoral medial rotation and/or adduction during the loading phase of running and/or other weight bearing activities has been proposed as a risk factor for lower limb disorders, such as iliotibial band syndrome [45], patellofemoral pain syndrome [46,47] and anterior cruciate ligament rupture [48]. Exercises aimed at “correcting” this movement pattern are often focused on strengthening the hip abductor and lateral rotator muscles while minimizing the activation of medial rotator muscles [20,49]. Numerous cross-sectional studies have shown an association between hip abductor and/or lateral rotator strength and dynamic knee valgus, however the summary evidence from prospective studies that these factors contribute to an increase in knee injury risk remains inconclusive [50]. 

Here, we would like to point to two aspects in the use of corrective exercises aimed at strengthening the lateral rotator muscles. Firstly, the biomechanical framework that is currently used to link hip lateral rotation strength deficit to knee valgus occurrence during running and, secondly, the often non-intuitive biomechanics of functional, weight bearing corrective exercises. 

Hip biomechanics was introduced as a “generic” risk factor for lower limb injury during the first decade of this century, and the role of the hip lateral rotator muscles has received great attention since then [50]. However, it seems that the link between hip lateral rotator strength deficit and excessive femoral medial rotation is often based on a misinterpretation of the running kinetics. During the loading response phase of running, an angular displacement of the hip towards flexion, adduction and medial rotation is observed. Powers et al (2010), argued in theirhighly cited paper regarding the “Influence of Abnormal Hip Mechanics on Knee Injury”, that this “triplanar motion is caused by the external moments acting on the hip joint and is resisted by actions of the hip extensors, abductors, and external (lateral) rotators”. This rationale has led to several studies investigating the role of lateral rotator and abductor muscles in preventing the “tendency” of the femur to medially rotate and has been used in support of corrective exercises aimed at strengthening these muscles. 

This argument is based on the common error of assuming that the direction of change in position or joint angle also reflects the direction of the external force/moment. Similar to a car breaking, in which the resultant force applied on the car is in the opposite direction to the displacement of the car, the angular displacement of the hip in the loading response phase may well be opposite to the direction of the external moment. In fact, although there seems to be a variation in the shape of the hip transverse moment throughout the stance phase across studies, a hip external moment of lateral rotation seems to be present throughout the loading response phase of running [51,52,53]. Experimental evidence also shows that the magnitude of these moments in the transverse plane is small (0.30 Nm/kg) relative to the capacity of the hip to generate torque in the transverse plane (1.0 to 2.7 Nm/kg) [54], which suggests that the “excessive” hip medial rotation that has been considered a risk factor for lower limb injuries is not caused by poor lateral rotator hip strength failing to resist a medial rotation of the femur.

Erroneous assumptions have also been made when prescribing corrective exercises. Movement based, or so called “functional” exercises, are continuing to grow in popularity, but the way targeted muscles are affected by these exercises is not always obvious. For example, the side-stepping exercise is widely used in training and rehabilitation protocols. It is performed in a squatted posture with the person moving laterally with a looped resistance band around the lower limbs. This exercise is typically used to strengthen the hip abductor and hip lateral rotator muscles [55,56,57], and recommendations have been made to place the resistance band around the forefeet to emphasize the control and recruitment of hip lateral rotator muscles [55,57]. However, based on a static mechanical analysis of this exercise, it would appear—at least for the unconstrained open kinetic phase characterized by a single-leg stance—that the elastic band creates a torque of lateral rotation around the hip, which would require the activation of hip medial—not lateral—rotator muscles as agonists to perform the exercise properly.

Biomechanics is a science with solid applications to human movement screening, rehabilitation, and exercise. However, if not carefully assessed, and if conclusions are drawn based on intuition, mistakes can be made and perpetuated, creating frameworks that may lead research and clinical efforts into a wrong direction. Future studies, exploring the mechanics of human movement in different populations and including joint power analyses may help to understand better the role of muscles in controlling joint kinematics during running [58]. In addition, more studies are needed addressing the muscular control of joint movement of commonly used corrective exercises, as much of the literature relies solely on the assessment of muscle activation.

## 6. The Importance of Corrective Exercise for Better Health and Superb Performance

### Highlighted by Antonino Bianco

The lack of motor coordination may significantly impair people’s lifestyle and life expectancy [59], plus this issue has been investigated so often in the last decade. Inside this scenario perfectly falls the “corrective exercise” approach that seems to be one of the major candidates for becoming a primary determinant for functional movement improvements and injury risk prevention. Madadi-shad et al. [60] recently published an interesting paper investigating the effect of a corrective exercise (CE) program on gait kinetics and muscle activities in older adults with both low back pain and pronated feet. The research team administered a proper training protocol of corrective exercise based mainly on stretch training and band exercises. The CE program, administered for 42 training sessions, improved walking performance, and provided lower pain and lower low-back-pain related disabilities [60]. On the other side, Jafari et al. [61] investigated CE training’s effects in Firefighters’ Functional Movement Screen (FMS) Scores. In the way I see it, this manuscript is of interest because it points out the importance of the functional fitness assessment in order to plan and administer a proper CE program [61,62,63,64]. In more detail, a number of 524 active firefighters completed all FMS testing procedures, and consequently, the sample has been clustered according to the obtained scores. The participants voluntarily participated in a dedicated CE program that lasted for eight weeks and was designed according to the National Academy of Sports Medicine (NASM) guidelines [61]. The authors reported improvements within the experimental group and highlighted the importance of the correct performance of fundamental functional movements to develop firefighters’ physical fitness. In that case, the importance of a correct “dosage” of exercise, in terms of volume, intensity, typology, and technique, appears clearly. An Italian team recently explored the effect of a 20-week corrective exercise program on functional movement patterns in youth elite male soccer players [33]. The research has been carried out by Campa et al. in 2019 and refers to a high prevalence of asymmetric movements during FMS administration in youth elite soccer players (Allievi Nazionali) [33]. Like the previous manuscripts, also, in this case, the authors administered a proper CE program that readers and football professionals can easily found within the manuscript, in Table 1 of ref [33]. As a result, the research shows FMS score improvements, in addition to emphasizing the possible reduction of asymmetries and dysfunctional movements [33].

The brief commentary of the manuscript mentioned above highlights how different are CE protocols (due to the correct personalization of the training) and how similar the results are in terms of FMS better scoring, better gait control, and dysfunctional movement reduction. 

In conclusion, the importance of CE in injury prevention and posture-related disorders for professional sports players appears clear. CE is a crucial component of the annual training periodization plan of many sports and recreational/fitness activities, and it deserves more attention from the scientific community, mainly from sports scientists.

## 7. Corrective Therapeutic Exercise on Postural Spine Curvature Disorders

### Highlight by Giuseppe Musumeci

Corrective exercise usually is used also to prevent, correct, or reduce postural spine curvature disorders. There are three principal types of spine curvature disorders, lordosis, also called swayback, kyphosis, and scoliosis characterized by a lateral curve to the spine (S-shaped or C-shaped). Structural alterations to the spine may lead to functional disturbances that, in turn, cause pain, inflammation, muscle weakness, nervous system stimuli disorders, and injuries. Spine deviations are classified as “paramorphism” with ligamentous, tendon and muscle involvement (reducible), and “dysmorphisms” with bone involvement (nonreducible). In a recent interesting study by Ceballos Laita et., al [65], the authors determined the effects of corrective, therapeutic exercise on patients diagnosed with adolescent idiopathic scoliosis (AIS). A spinal deformity is called “scoliosis” when the spine shows a frontal deviation with a spinal curvature (Cobb’s angle) of 10° or more, and it is associated with rotation of the vertebral bodies. Specifically, AIS is the most common spinal deformity and has high diagnostic relevance in the pediatric population, mostly during adolescence. The typical signs of AIS are chest deformities, protuberances, and asymmetries. The outcomes of this review paper appear to point out the positive effects of AIS management with therapeutic exercise based on the Schroth method or stabilization exercises. Therapeutic exercise decreases symptoms and improves function, vertebral angles, and trunk asymmetries. Instead, in another recent study by Alanazi et al., [66] they studied the effects of stabilization exercises on back pain, disability and quality of life in adults with scoliosis. The adult scoliosis (AS), is the most common spinal deformity in adults. Back pain is the main symptom leading patients to seek medical consultation. Stabilization exercises, as reported in this study, have shown to be effective in reducing back pain, disability and improving quality of life in adults with idiopathic scoliosis. However, this review paper highlights the paucity of the literature examining the effect of exercise on back pain in adults with scoliosis. Until now it is not possible to describe the ideal moment for the intervention or the number of weekly sessions and the duration of each session. Therefore, further experimental studies on corrective therapeutic exercise on postural spine curvature disorders are necessary to strengthen the current results in the literature. It is desirable to guarantee a better methodological quality on therapeutic exercise that measures clinical and imaging outcomes to obtain conclusive and satisfactory results in order to improve the quality of life of these patients. 
